# Cellenium—a scalable and interactive visual analytics app for exploring multimodal single-cell data

**DOI:** 10.1093/bioinformatics/btad349

**Published:** 2023-06-01

**Authors:** Carsten Jahn, Mahmoud Ibrahim, Jannis Busch, Qiong Lin, Himanshu Manchanda, Hagen Mohr, Dan Plischke, Helge G Roider, Andreas Steffen

**Affiliations:** Scientific Solutions, Bayer AG, Berlin 13353, Germany; Biomedical Data Science, Bayer AG, Wuppertal 42113, Germany; Scientific Solutions, Bayer AG, Berlin 13353, Germany; Scientific Solutions, Bayer AG, Berlin 13353, Germany; Biomedical Data Science I, Bayer AG, Berlin 13353, Germany; Scientific Solutions, Bayer AG, Berlin 13353, Germany; Scientific Solutions, Bayer AG, Berlin 13353, Germany; Biomedical Data Science I, Bayer AG, Berlin 13353, Germany; Scientific Solutions, Bayer AG, Berlin 13353, Germany

## Abstract

**Summary:**

Multimodal single-cell sequencing data provide detailed views into the molecular biology of cells. To allow for interactive analyses of such rich data and to readily derive insights from it, new analysis solutions are required. In this work, we present Cellenium, our new scalable visual analytics web application that enables users to semantically integrate and organize all their single-cell RNA-, ATAC-, and CITE-sequencing studies. Users can then find relevant studies and analyze single-cell data within and across studies. An interactive cell annotation feature allows for adding user-defined cell types.

**Availability and implementation:**

Source code and documentation are freely available under an MIT license and are available on GitHub (https://github.com/Bayer-Group/cellenium). The server backend is implemented in PostgreSQL, Python 3, and GraphQL, the frontend is written in ReactJS, TypeScript, and Mantine css, and plots are generated using plotlyjs, seaborn, vega-lite, and nivo.rocks. The application is dockerized and can be deployed and orchestrated on a standard workstation via docker-compose.

## 1 Introduction

The rapidly expanding volume of single-cell omics data in biomedical research not only represents an invaluable resource for unraveling biological mechanisms underlying cellular changes during for instance normal development and disease but also presents an unprecedented challenge for data curation, data storage, and visualization. Several single-cell browsers have been developed with a focus on visualizing transcriptomics data (Zappia and Theis 2021), but often with limited applicability to multimodal data and mostly without across-dataset search functionality, semantic metadata integration, and performant analysis capabilities and a focus on user experience. Our new application, Cellenium, is a full-stack scalable, powerful visual analytics web application, which allows for ultra-fast and detailed exploration of integrated single-cell data within individual studies and across datasets. Cellenium puts a particular emphasis on a high-quality user experience as well as on a data model that allows for efficient searching and browsing of a large number of integrated datasets. Cellenium focuses on three main scRNA-Seq analysis use cases: (i) comprehensive expression analysis across cell types within a single study, (ii) comparative expression analysis across multiple studies, and (iii) cross-study marker gene search. To enable these use cases in an effective way, we designed Cellenium with the primary goal of providing fast responses to user requests. Next to scRNA-Seq data, Cellenium allows for the integration of CITE- and ATAC-Seq data sets (Bredikhin 2022).

## 2 Materials and methods

Cellenium consists of a central Postgres database for hosting all expression- and metadata, a Postgraphile-based GraphQL API layer, as well as a reactjs/typescript GUI, all of which are dockerized and orchestrated using docker-compose (for details see supplementary material). We strived to keep the application architecture simple and designed Cellenium in a way that it does not rely on any particular cloud services. In that way, Cellenium can also be deployed on on-premise hardware. The PostgreSQL database and its PostGraphile-based GraphQL API layer are the only server-side components. Database functions implement core API features like (optional) study access authorization, aggregating expression data on gene and cell-type level, and even handle calculations in database background jobs. During the installation, the docker-compose stack builds custom docker images for PostgreSQL 15 and PostGraphile.

Single-cell omics studies to be integrated need to be provided in the AnnData format (h5ad) or a multi-omics variant thereof (h5mu). Cellenium expects metadata attributes of a study to be added to the unstructured annotation field of AnnData files (uns-dictionary). These include title, description as well as species (NCBI taxonomy), disease annotation (NCBI MeSH), or tissue types (NCIT) of the study. Further metadata such as previously annotated cell types can also be provided. In addition, Cellenium uses CellO (Bernstein 2021) to provide standardized cell type annotations for each individual cell. To aid with the creation of suitable import data files, Cellenium provides python utility functions that add data in the AnnData structure and validate the file. Example jupyter notebooks, which download and prepare multiple studies for import to Cellenium, are provided in Cellenium’s github repository. For the demo installation, the notebooks are executed in headless mode by Make, which is configured through a Makefile that is part of the Cellenium demo setup. The notebooks can also easily be executed interactively as well, and thus serve as a starting point to add custom data sets. The database schema contains tables for common reference data such as genes and ontology concepts as well as study-specific data. The study data define all genes and cells that are present in the study as well as corresponding expression levels. As scRNA expression data are a sparse matrix, we defined a suitable storage-efficient data structure and organized data in a gene-centric approach that allows effective querying of the data.

**Figure 1:**
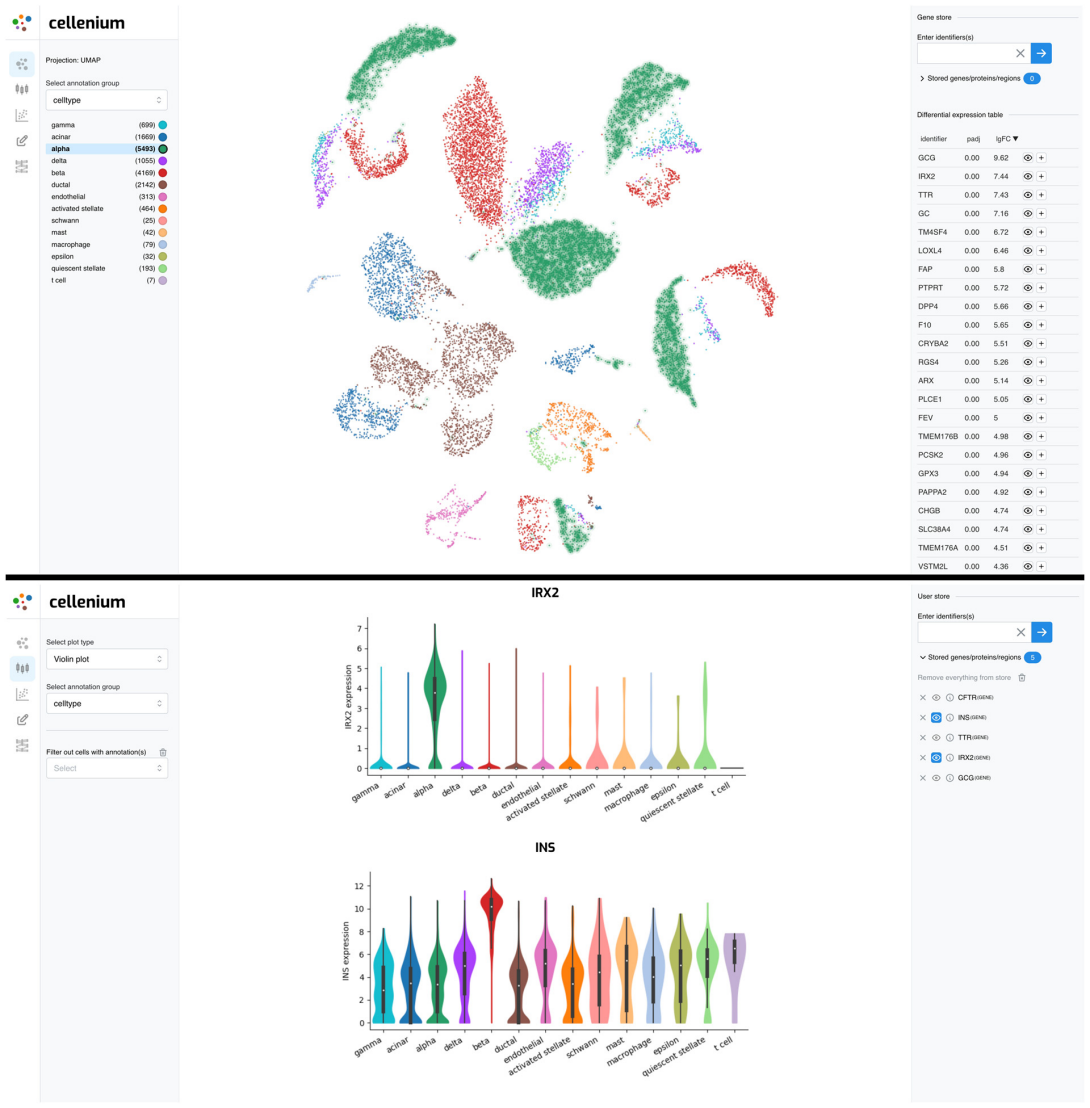
In the interactive cell type exploration mode of Cellenium, users can explore differentially expressed genes for each cell type of a study (see upper part of the figure). By adding genes to the user gene store users can be performed further analyses in other sections (e.g. violin expression plots see bottom part of the figure).

In the single study analysis section (use case 1), users can interactively identify marker genes of predefined cell types. The selection of cell types is possible either on the provided annotations on the side panel of the GUI or on the UMAP-based projection plot by clicking on the corresponding cells directly (see Fig. 1). For efficiency reasons, Cellenium precalculates differential gene expressions between each annotated cell type and all other cells of the study. In that way, the user receives immediate feedback when selecting a cell type and can quickly identify respective differentially expressed genes for the selected cell type. In the case of single nuclei ATAC-Seq and CITE-Seq data, the user can directly link genes to genomic regions or antibodies respectively. Individual mRNA or protein expression patterns across all cell types can be further assessed using violin or projection plots generated using seaborn (https://seaborn.pydata.org/) and plotlyjs (https://plotly.com/javascript/). In the co-expression view, pairwise gene expression scatter plots can be generated. In addition, correlated genes can be identified transcriptome wide for any given gene of interest. Additionally, the user can explore and store self-defined cell types using an interactive cell-marker definition widget. These cell types can then be interactively visualized on the projection plots. Through this functionality, the user can for instance analyze how user-defined cell types relate to pre-annotated cell types or identify differentially expressed genes for the defined cell types. To allow for a visual comparison of the relationships between different cell type annotations within a study, we developed an interactive Sankey plot implemented using the javascript library nivo.rocks (https://github.com/plouc/nivo).

The cross-study analysis section (use case 2) enables the comparison of gene expression levels in the CellO-based predicted standardized cell types across different studies. The expression levels are visualized by vega-lite (Satyanarayan 2017) using a dotplot visualization type e.g. known from Seurat (Hao *et al.* 2021) and scanpy (Wolf 2018). This visualization provides a concise overview of expression levels in aligned cell types of different tissues.

The third section (use case 3) is designed for conducting marker gene searches across all integrated studies. Here, users can input genes of interest (e.g. insulin) and analyze in what cell types these genes are specifically expressed within their corresponding study (in the case of insulin the users would for example find pancreatic beta-cells). Since differential gene expression is pre-calculated during the data ingestion, results are obtained in real-time without delay. Differential expression is visualized using inline volcano plots for quick inspection.

## 3 Conclusion

In this publication, we have described Cellenium, a scalable, interactive, and highly performant web application for multimodal single-cell data storage, retrieval, and analysis. Cellenium provides a flexible and intuitive user interface for common integrative analysis and visualization tasks and enables deep analysis of integrated single-cell studies. We believe that the simplicity of the application design might enable also non-bioinformaticians to gain detailed insights from vast single-cell sequencing studies through elaborate analysis techniques.

## Supplementary Material

btad349_Supplementary_Data
