# Supplementary material for: Cellenium—a scalable and interactive visual analytics app for exploring multimodal single-cell data
Source: Bioinformatics. 2023 Jun 1;39(6):btad349. doi: 10.1093/bioinformatics/btad349 (PMC10257576; doi:10.1093/bioinformatics/btad349)

# Supplementary Information

Cellenium is a FAIR and scalable interactive visual analytics app for multi-modal single-cell data. It allows to:

- Organize and semantically find scRNA studies with ontologized metadata for tissues and diseases
- explore cell types and other cell annotations in UMAP space
- find differentially expressed genes based on clusters of annotated cells
- view the expression of a single gene (or a few selected genes) in the UMAP plot or as grouped violin plots
- draw co-expression plots for pairs of genes, explore the cell types contained in the plots
- add new cell annotations based on plot selections, see differentially expressed genes for a selected group of cells
- find genes which expression is highly correlated to a query gene
- find marker genes in all imported studies and qualitatively compare gene expression across studies.

## Github repository

<https://github.com/Bayer-Group/cellenium/>

## Youtube showcase

<https://youtu.be/U71qIK-Mqlc>

## Application architecture


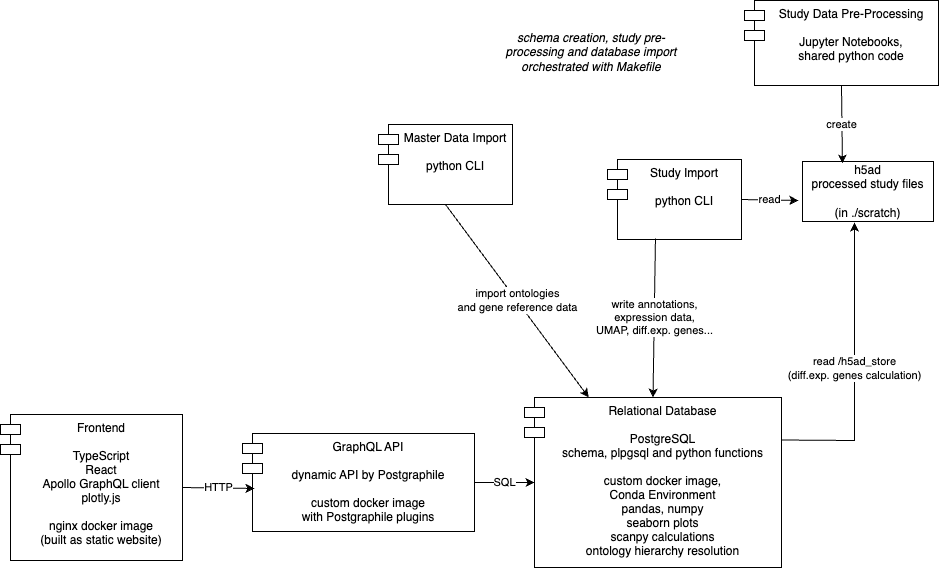


## Database schema


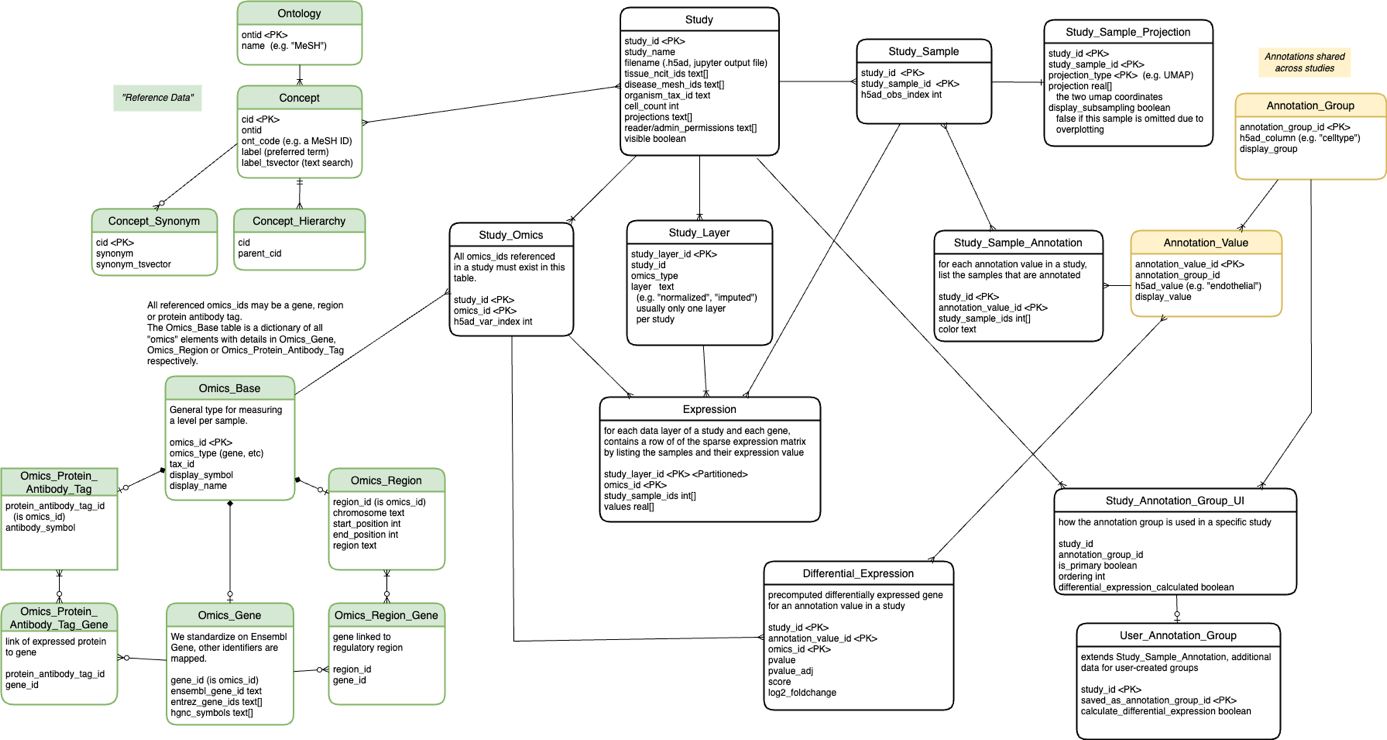

Supplement: btad349_Supplementary_Data [file btad349_supplementary_data.docx]
